# Condensate interfaces can accelerate protein aggregation

**DOI:** 10.1016/j.bpj.2023.10.009

**Published:** 2023-10-13

**Authors:** Chang-Hyun Choi, Daniel S.W. Lee, David W. Sanders, Clifford P. Brangwynne

**Affiliations:** 1 Department of Chemical and Biological Engineering, Princeton University, Princeton, New Jersey; 2 Omenn-Darling Bioengineering Institute, Princeton University, Princeton, New Jersey; 3 Howard Hughes Medical Institute, Chevy Chase, Maryland

## Abstract

Protein aggregates, formed from the assembly of aberrant, misfolded proteins, are a hallmark of neurodegenerative diseases. Disease-associated aggregates such as mutant Huntingtin polyQ inclusions, are typically enriched in p62/SQSTM1, an oligomeric protein that binds to and sequesters aberrant proteins. p62 has been suggested to sequester proteins through formation of liquid-like biomolecular condensates, but the physical mechanisms by which p62 condensates may regulate pathological protein aggregation remain unclear. Here, we use a light-inducible biomimetic condensate system to show that p62 condensates enhance coarsening of mutant polyQ aggregates through interface-mediated sequestration, which accelerates polyQ accumulation into larger aggregates. However, the resulting large aggregates accumulate polyubiquitinated proteins, which depletes free p62, ultimately suppressing further p62 condensation. This dynamic interplay between interface-mediated coarsening of solid aggregates and downstream consequences on the phase behavior of associated regulatory proteins could contribute to the onset and progression of protein aggregation diseases.

## Significance

p62/SQSTM1 is a protein characteristic of aggregates found in diverse neurodegenerative diseases. Its capacity to form liquid-like condensates has been suggested to aid in the sequestration of aggregation-prone proteins; however, the biophysics of p62 condensation and its impact on neurodegeneration-associated protein aggregates are poorly understood. Here, we uncover a key role for the surface of p62 condensates in the coarsening of solid-like Huntingtin aggregates. p62 condensate surfaces recruit smaller aggregates, which subsequently coarsen and generate larger aggregates, but this process eventually hinders further p62 condensation. These findings suggest an unexpected interplay between liquid-like biomolecular condensates and solid-like protein aggregates, which could play a role in the progression of neurodegenerative pathologies.

## Introduction

An enduring challenge of modern cell biology is understanding the mechanisms by which individual proteins form intracellular assemblies governing cell physiology and disease. From small complexes of several proteins and nucleic acids to large collectives of more than tens of thousands of biomolecules, this emergent organization occurs across a wide range of length scales. Cells dynamically generate assemblies to regulate essential functions such as transcription (1), RNA processing (2), DNA repair (3), and signal transduction (4). However, large, persistent biomolecular assemblies are also defining features of pathological diseases such as Alzheimer’s, Huntington’s, and amyotrophic lateral sclerosis (5,6,7,8).

Among mechanisms driving large-scale biomolecular organization, phase separation and associated phase transitions have been proposed to influence various cellular processes. Phase separation is thought to play a central role in driving the emergent self-assembly of dozens of liquid-like biomolecular condensates, including nuclear bodies such as nucleoli and Cajal bodies, cytoplasmic bodies such as stress granules and P bodies (9,10,11,12), as well as precursors to gel-like assemblies typical of pathological aggregates (13,14,15,16,17). Proteins that undergo intracellular phase separation into condensates typically require high interaction valence to form dynamic interaction networks (18,19,20), which can be achieved through oligomerization domains, coupled to “sticky” intrinsically disordered regions, as well as traditional folded domains that mediate specific substrate binding (e.g., with RNA and DNA) (19,21).

The oligomerization that typically drives liquid-like condensation also has key relevance to the formation of various other types of assemblies, particularly the formation of solid-like protein aggregates. Aberrant, often mutated proteins associated with neurodegeneration such as tau, a-synuclein, and Huntingtin polyQ misfold and avoid protein degradation mechanisms, clustering into oligomers, and eventually large, ubiquitin-rich aggregates (5). An oligomeric protein, p62/SQSTM1 (p62), is recognized as a canonical marker of ubiquitinated aggregates for autophagic degradation (22,23). More recent studies have suggested that p62 itself can undergo phase separation to form protein condensates (24,25). Its self-oligomerization PB1 domain and its ubiquitin substrate-binding UBA domain are thought to operate in tandem to drive p62 condensation both in cells and in vitro (24,26). Indeed, polyubiquitin, a marker for protein degradation, among its many roles, is a p62 substrate that may serve as a multivalent scaffold, promoting p62 oligomers to further assemble into mesoscale condensates (24,27). Although p62 condensates have been traditionally described as sites to sequester diffuse misfolded proteins (28), the changes in material properties and size of p62-associated aggregates suggest a more complex relationship between p62 phase behavior and pathological aggregation.

One potentially interesting aspect of this interplay is that p62 condensates have been found to be liquid-like, whereas protein aggregates are generally more solid-like (5,24). Interactions between liquids and solids are ubiquitous in nonliving matter (e.g., water condensation on a cold glass) and examples have also been recently identified in the complex environment of the cell. These include the association of ribonucleoprotein condensates with endoplasmic reticulum membranes (29), the wetting of proteins on microtubules (30,31), and the decoration of MEG-3 clusters onto P granules (32). In each of these examples, the interfacial tension between the liquid and solid surfaces will play a key role in the wetting behavior (33) and can give rise to capillary forces that can pull together micrometer-scale structures (34,35,36,37,38,39). However, the extent to which similar effects may be at play between condensates and solid-like protein aggregates, which form and coarsen in neurodegenerative diseases, remains unclear.

Here, we show that synthetic p62 condensates sequester and coarsen protein aggregates in living cells. Using an engineered light-inducible scaffold to controllably mimic native p62 oligomerization, we generated de novo p62 condensates in the presence of mutant Huntingtin polyQ (PolyQ) aggregates implicated in Huntington’s disease. We found that the interfaces of these newly formed condensates sequestered PolyQ aggregates, which unexpectedly led to the aggregates rapidly coarsening upon condensate dissolution. However, large, highly ubiquitinated PolyQ aggregates strongly recruited p62 oligomers and reduced free p62 throughout the cell, inhibiting subsequent p62 condensation. Our findings on PolyQ aggregate coarsening through p62 condensation, and the inhibition of condensation by aggregate ubiquitination, underscore a dynamic interplay between phase separation and protein aggregation in living cells.

## Materials and methods

### Plasmid construction and transduction

Plasmid constructs were generated by In-Fusion protocols (Takara Bio) and expressed by lentiviral transduction with FuGENE HD Transfection Reagent (Promega). Please refer to the supporting material for specific details.

### Cell culture

HeLa CCL-2 (ATCC), U2OS (kind gift from Mark Groudine, Fred Hutchinson Cancer Research Center), and Lenti-X 293-T (Takara Bio) cells were cultured in DMEM (Gibco) with the addition of 10% FBS (Atlanta Biological) and 1% streptomycin and penicillin (Gibco). Cells were incubated at 37°C in 5% CO_2_. One day before imaging, cells were washed with Dulbecco's phosphate-buffered saline (Thermo Fisher), trypsinized, and replated in 96-well glass-bottom imaging plates (Cellvis) coated using a PBS solution with 0.25 mg/mL fibronectin (Thermo Fisher).

### Immunofluorescence and western blotting

Commercial primary antibodies specific to the proteins of interest, along with secondary antibodies, were utilized for performing both methods. Please refer to the supporting material for specific details.

### Live-cell imaging, z stack

Live sample imaging was performed using a Nikon A1 laser-scanning confocal microscope with a Plan Apo 60×/1.4 oil immersion objective (Nikon), and a Zeiss LSM 980 laser-scanning confocal microscope with a Plan-Apochromat 63×/1.4 oil immersion objective (Zeiss). On both microscopes, plates were kept at a 5% CO_2_ and 37°C humid environment using stage incubators, and, on the LSM 980, an additional environmental chamber. Excitation of mCherry, Hoechst dye, EGFP, miRFP670, and Alexa Fluor 568 were performed using 405-, 488-, 561-, and 639-nm lasers. 3D images were gathered with optical sectioning of 250 nm for the LSM980 and optical sectioning of 420 nm for the Nikon A1. For light-inducible droplet condensation experiments, 488-nm laser was activated to oligomerize the sspB-fused construct to the iLID-fused core to observe light-inducible p62 droplet condensation and coarsening.

### Estimating live-cell protein concentrations and measuring phase diagrams

Protein concentration estimates for EGFP-polyQ74, EGFP-polyQ31, mCherry-p62, mCherry-p62ΔPB1, mCherry-p62ΔUBA, sspB-mCherry-p62ΔPB1, and iLID-EGFP-ft were measured using settings on the Nikon confocal A1 microscope matching conversions from absolute concentration values to fluorescence intensity for EGFP and mCherry fluorophores utilizing fluorescence correlation spectroscopy (32). Phase diagrams were obtained by measuring the fluorescence intensities of sspB-mCherry-p62ΔPB1 iLID-EGFP-ft in the cytoplasm of HeLa cells and U2OS cells co-expressing both corelet constructs. An area of ∼10 *μ*m^2^ was selected for estimation of the cytoplasmic fluorescent intensity before the nucleation of any light-inducible corelets. A 5-min blue light activation cycle was performed according to the live-cell imaging protocols for condensation and cells were determined to either be capable of condensation and phase separation or lacking in condensation with no phase separation. The cytoplasmic fluorescent intensities were converted to protein concentrations through the FCS-derived conversion and values such as core concentration, the concentration of 24-mers of iLID-EGFP-ft and valence, the ratio of concentrations of 24-mer iLID-EGFP-ft and sspB-mCherry-p62ΔPB1, and estimating the average valence of each core in a specific cell cytoplasm were measured as axes of the phase diagrams. For the phase diagrams evaluating the impact of polyQ aggregation on cell-wide inducible p62 condensation, a similar approach was taken, except cells expressing miRFP-polyQ74 aggregation or lack of aggregation were distinguished either as displaying cell-wide condensation or no cell-wide condensation. Cell-wide condensation was characterized as a lack of circular p62 droplets nucleated throughout the cytoplasm.

### Fluorescence recovery after photobleaching

HeLa cells expressing mCherry-p62, EGFP-polyQ74, mCherry-p62ΔPB1, and iLID-EGFP-ft constructs in various combinations were photobleached with the respective 488- and 561-nm lasers matching the excitation of the fluorescent proteins fused to the construct of interest and allowed to recover for 120–125 s. All experiments were performed on a Nikon confocal A1 microscope, at the humidified conditions kept constant across all experiments. A circular region of interest of ∼1.5-*μ*m diameter was photobleached and the fractional recovery of the fluorescence intensity within this region was measured, normalized to the intensity in the first frame pre-bleach.

### Condensation/dissolution experiments

HeLa cells expressing p62 corelets and polyQ aggregates were imaged with a Zeiss LSM 980. Cells with accumulation of small polyQ aggregates and p62 corelet expression ratios of sspB-mCherry-p62ΔPB1 to iLID-ft to induce condensation were selected for condensation-dissolution cycling. As part of the condensation cycle, 561- and 488-nm lasers were used to image both sspB-mCherry-p62ΔPB1 and EGFP-polyQ. The duration of blue light exposure was 30 min after the live-cell imaging intervals of activation, a sufficient duration of time for blue 488-nm light to oligomerize the iLID-EGFP-ft 24-mers to the previously un-bound mCherry-p62ΔPB1 and induce robust p62 droplet condensation, coarsening, and polyQ aggregate recruitment. For the dissolution cycles, the blue light was deactivated, and dissolution imaged with the remaining 561-nm laser for a period of 15 min, far longer than required to observe dissolution of the induced p62 droplets. These pairs of condensation-dissolution cycles were performed a total of three times, followed by one final round of condensation, and data were analyzed from the first frame of each new condensation cycle.

### Image analysis

Measurements of individual aggregate volumes, aggregate areas, p62 condensate radii, and general segmentation were performed using Fiji (ImageJ 1.52p) and MATLAB (Mathworks) software. For 2D and 3D analyses, cells were individually cropped by hand, ensuring the entirety of the cytoplasm was captured within each crop. For 3D analyses, the .tif files containing the z stack data for each cell were then analyzed using MATLAB, where the frame with the maximum mean cytoplasmic brightness was selected to estimate the mean and standard deviation of intensity required to threshold the cytoplasm. In both 2D and 3D analyses, a selected region within the cytoplasm lacking dense condensates or aggregates was analyzed to select a threshold as the mean of the cytoplasmic intensity plus 2.5 standard deviations, applied to all relevant frames. To distinguish aggregates well above the diffraction limit through segmentation, a minimum 2D area threshold of 0.116 *μ*m^2^ or 16 pixels was enforced and, for 3D volumes, a threshold of 0.116 *μ*m^3^ or 64 pixels. For 2D analyses, the regionprops function was used to generate statistics for the thresholded objects, and, for 2D areas, the regionprops3 function was utilized. For complementary cumulative distribution functions (CCDF) of aggregate size, cumulative distribution functions were calculated for the aggregate size distributions of each cellular cytoplasm and then averaged, with an SE measurement.

## Results

### Inducing oligomerization of p62 generates ubiquitin-sensitive condensates in living cells

**Figure 1:**
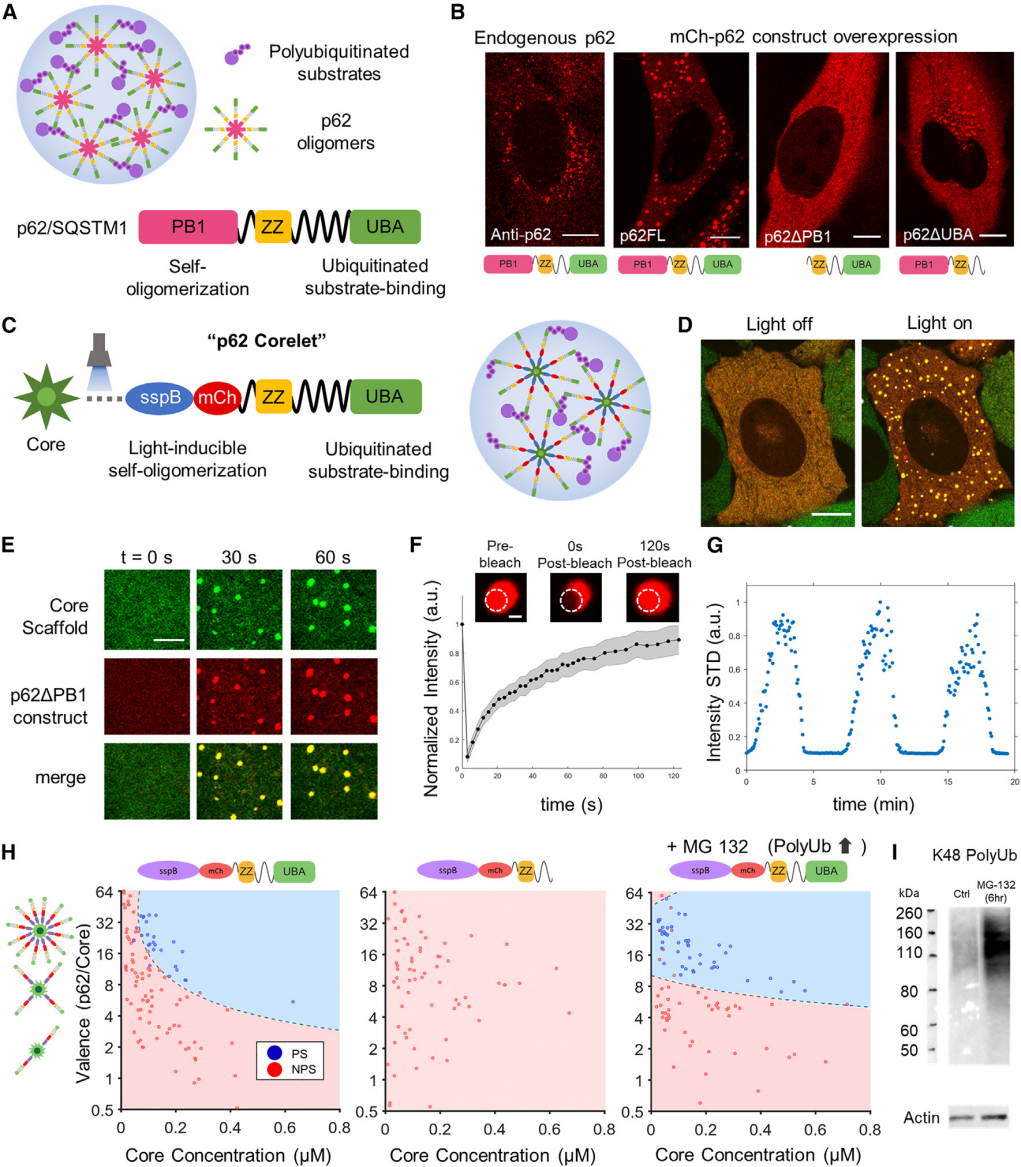
Engineering intracellular light-inducible p62 condensates. (*A*) Simplified diagram of p62 protein labeling self-oligomerization N-terminal PB1, ubiquitin-associated C-terminal UBA, and ZZ domains. (*B*) Images of endogenous p62 compared to mCherry-p62 construct overexpression for full-length p62 and truncations, p62ΔPB1 and p62ΔUBA. (*C*) Substitution of self-oligomerization PB1 domain with multivalent scaffold corelets. (*D*) De novo p62 condensates are inducible in the cytoplasm upon 488-nm stimulation. (*E*) Synthetic p62 condensates nucleating and coarsening during light activation. (*F*) FRAP of synthetic p62 condensates. Error bars are mean ± SE for 13 condensates. Scale bar, 1 *μ*m. (*G*) Changes in fluorescent intensity standard deviation across multiple cycles of light activation and deactivation. (*H*) Phase diagrams of oligomeric valence to concentration for constructs with UBA domain, without UBA domain, and with UBA domain and 10 *μ*M MG-132 treatment for 6 h. (*I*) Western blot of K48 polyubiquitin chains for cells with MG-132 treatment and nontreated control. Scale bars for all non-FRAP images, 5 *μ*m.

We first characterized the domain-specific phase behavior of p62 with the goal of developing an experimentally tractable system for studying interactions between de novo p62 condensates and intracellular aggregates (Fig. 1 *A*). Overexpression of a fluorescent mCherry-p62 fusion construct in HeLa cells gave rise to cytoplasmic p62 condensates, which are observed to undergo liquid-like coalescence (Fig. S1 *A*). To evaluate the material state of these p62 condensates, we performed fluorescence recovery after photobleaching (FRAP). We observed rapid but incomplete recovery, consistent with a partially liquid-like character (Fig. S2 *B*). Cells expressing a p62 construct with a deletion of the self-interacting oligomeric PB1 domain lacked the distinct large condensates observed in the overexpression of full-length p62. Deletion of the ubiquitinated substrate-binding UBA domain resulted in a smaller punctate pattern resembling endogenous p62 (Fig. 1 *B*). Although p62ΔPB1 is unable to form assemblies, p62ΔUBA puncta lack a characteristic of liquid-like condensates to coarsen into larger structures as a function of expression. The necessity of both PB1 and UBA domains for condensation is consistent with previous studies (22,26) and holds across a wide range of protein expression (Fig. S2 *A*).

We next sought to quantitatively dissect the biophysical determinants of p62 phase separation using a well-controlled synthetic system. Given the importance of self-oligomerization in driving p62 condensation, we replaced the native PB1 domain with an optogenetic multimeric scaffold for light-activated oligomerization, known as the corelet system (40). Specifically, we replaced the PB1 domain with the optogenetic sspB protein domain, which, upon blue light activation, strongly interacts with an iLID domain-decorated 24-mer ferritin scaffold (Fig. 1 *C*). Upon co-expression of these two constructs in cells, we find that they colocalize in de novo p62 condensates, which nucleate rapidly upon light activation (Fig. 1 *D* and *E*). These synthetic p62 condensates exhibit liquid-like FRAP recovery as seen with p62 overexpression condensates but can be nucleated on demand and dissolved through repeated cycles of on-off blue light activation (Fig. 1 *F* and *G*). Another advantage of the inducible system is its utility in mapping intracellular phase diagrams (Fig. 1 *H*); this phase diagram displays the location of a given cell in phase space with respect to the valence (i.e., ratio of the core scaffold to p62ΔPB1 construct, y axis) and the concentration of the oligomeric unit (i.e., core concentration, x axis). We find that p62 phase behavior is characterized by a well-defined phase boundary (binodal) with condensation occurring with sufficiently high valence and oligomerized core concentration. Furthermore, phase separation was abrogated upon removal of the UBA domain, consistent with an essential role for ubiquitinated substrates in p62 condensation (24,27). To further test whether an excessive accumulation of ubiquitinated substrates affects p62 phase behavior, we treated the cells with a proteasome inhibitor, MG-132, inhibiting ubiquitinated protein degradation (Fig. 1 *I*). MG-132 treatment resulted in a shift in the phase boundary reflecting an increased propensity for phase separation (Fig. 1 *H*). These findings underscore the utility of the p62 corelet system for studying functionally relevant p62 phase behavior in a quantitative and highly controllable intracellular system.

### Huntingtin polyQ as a model ubiquitinated aggregation system with continuous coarsening and robust p62 recruitment

**Figure 2:**
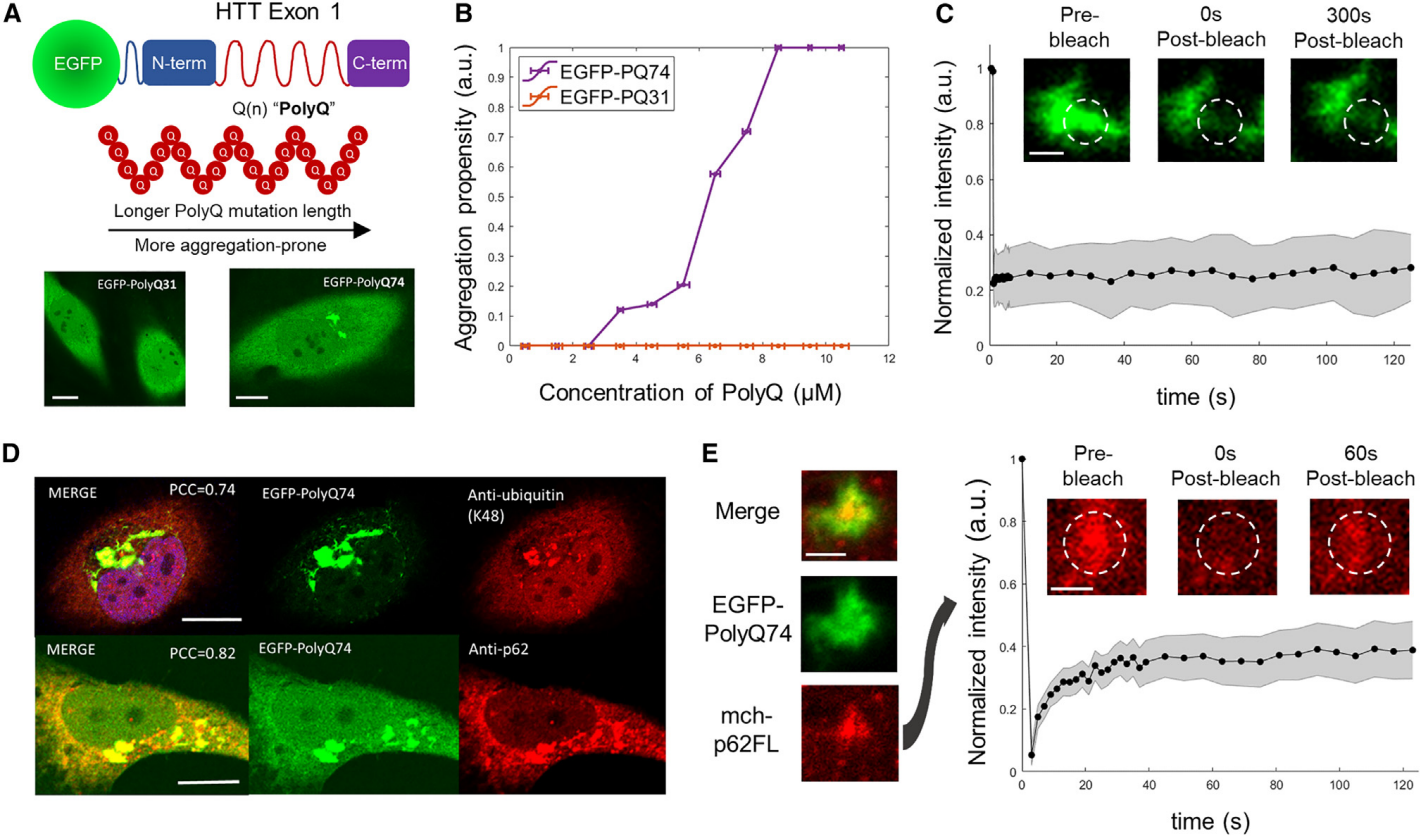
Mutant Huntingtin PolyQ as a model system for ubiquitinated aggregates with robust p62 co-localization. (*A*) Diagram of mutant Huntingtin Exon 1 fragment used as a model aggregation system. Images of live HeLa cells expressing EGFP-PolyQ31 compared to EGFP-PolyQ74 with a longer mutant repeat Q(n) domain. Scale bar, 5 *μ*m. (*B*) Graph of aggregation propensity versus total cytoplasmic concentration of PolyQ. The two curves are for HeLa cells expressing polyQ74 (purple) and HeLa cells expressing PolyQ31 (red) lacking aggregation propensity. (*C*) FRAP curve of EGFP-PolyQ74 aggregates with a recovery period of 2 min for nine aggregates. (*D*) Immunofluorescence for endogenous p62 and K48-linked polyubiquitin for a HeLa cell expressing PolyQ74 aggregates in the cytoplasm. Scale bar, 5 *μ*m. (*E*) FRAP curve of p62 co-localized with PolyQ aggregates for eight aggregates. Scale bars for all FRAP images, 1 *μ*m. Error bars are based on mean ± SE.

To study the interplay between neurodegeneration-associated aggregates and p62 condensates, we chose mutant Huntingtin Exon1, a protein directly associated with Huntington’s disease. The length of the repeat polyglutamine or polyQ tract is known to be a primary determinant of Huntingtin aggregation and disease progression (41,42,43,44). Consistent with polyQ length dependence, we observed no aggregation in cells stably transfected with a short EGFP-PolyQ31 construct but aggregation with the longer EGFP-PolyQ74 construct (Fig. 2 *A*). We determined that the aggregation propensity, defined as the likelihood of observing intracellular aggregates, was dependent on protein concentration for PolyQ74, whereas PolyQ31 failed to show any aggregation propensity across a wide range of concentrations (Figs. 2 *B* and S3 *A*). In contrast to the p62 condensates described previously, PolyQ74 aggregates display no recovery by FRAP, reflecting the solid-like properties of these aggregates (Fig. 2 *C*). The resulting aggregates continuously coarsen in the cytoplasm (Fig. S3 *B*), as shown in aggregate size distributions measured at different points in time (Fig. S4). We then characterized the cytoplasmic PolyQ74 (hereafter PolyQ) aggregates for both their ubiquitination and recognition by p62. We visualized endogenous p62 and K48-linked polyubiquitin in PolyQ aggregate-expressing cells and observed strong co-localization between p62, polyubiquitin, and PolyQ (Fig. 2 *D*), indicating that PolyQ aggregates are dense ubiquitinated inclusions recognized by p62. In cells expressing both overexpressed p62 and PolyQ aggregates, FRAP of p62 showed that a majority of co-localized p62 is immobile, with a small fraction of p62 species remaining mobile (Fig. 2 *E*). Huntingtin polyQ, with its capacity to form ubiquitinated aggregates with robust recruitment of p62, is thus a favorable system to study alongside inducible p62 condensation.

### p62 condensate interfaces can accelerate coarsening of PolyQ aggregates

**Figure 3:**
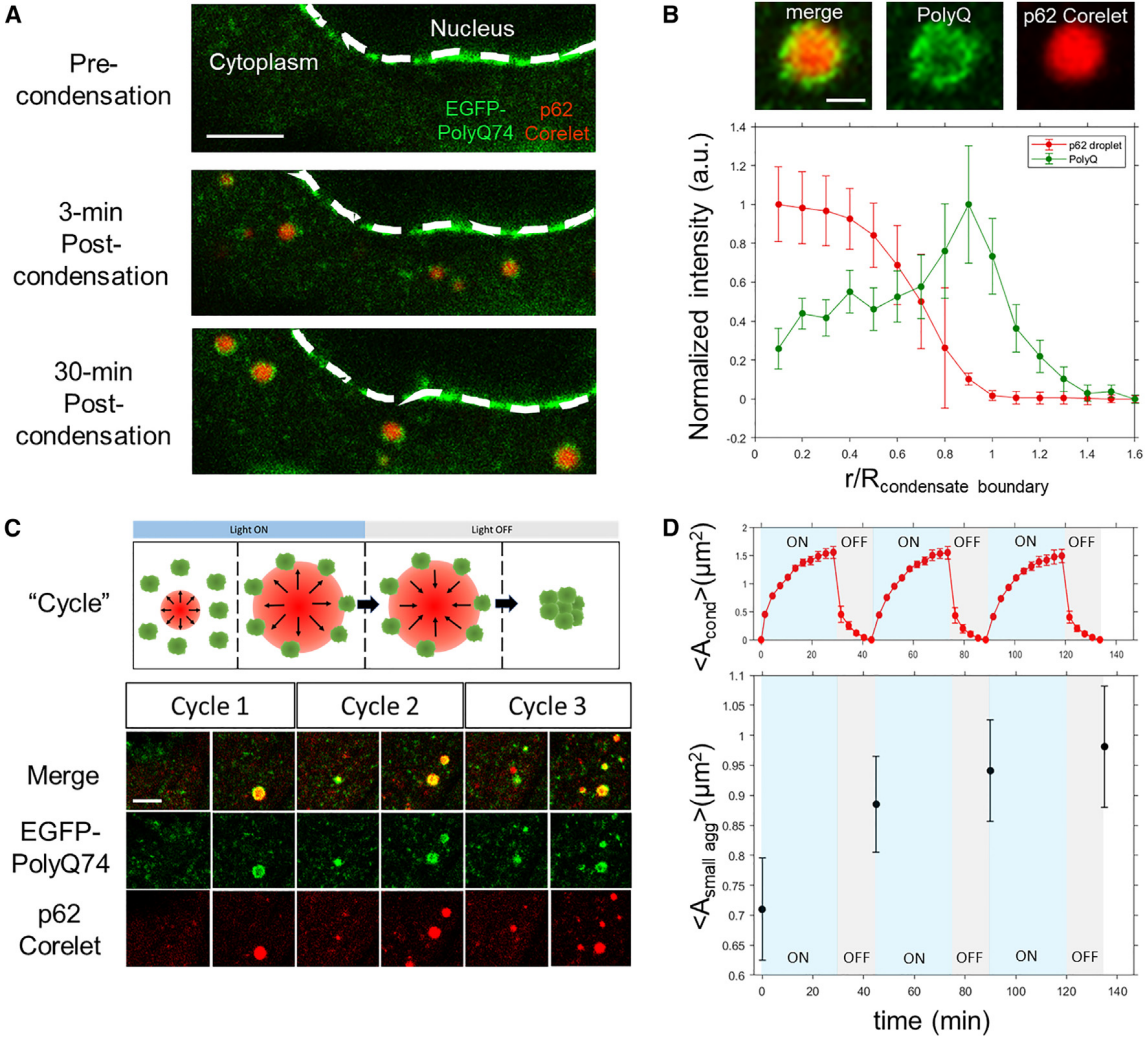
De novo p62 condensates preferentially cluster small PolyQ aggregates on their interfaces. (*A*) Live imaging of HeLa cells expressing EGFP-PolyQ74 aggregates and de novo p62 corelet condensates formed over the course of 30 min of blue light stimulation. Scale bar, 5 *μ*m. (*B*) Radial intensity plot of 29 condensates across 11 cells exhibiting aggregate accumulation at p62 condensate interfaces. Scale bar, 1 *μ*m. (*C*) Imaging HeLa cells exhibiting PolyQ aggregate accumulation on de novo p62 condensate peripheries, involving three cycles comprising 30 min of light activation for de novo condensation and 15 min of deactivation to allow for condensate dissolution. Scale bar, 1 *μ*m. (*D*) Quantification of the mean area of induced p62 condensates after three cycles of p62 condensation and dissolution for 10 cells. Quantification of the mean area of small aggregates (<5 *μ*m^2^) coarsened at the beginning and end of each cycle of condensation and dissolution. Error bars are based on mean ± SE.

We next utilized the light-activatable p62 condensate system in cells expressing the model PolyQ aggregates to interrogate the relationship between p62 condensation and PolyQ aggregation. Upon continuous light activation, we unexpectedly observed that some cells displayed small PolyQ aggregates on the surfaces of induced p62 condensates, forming ring-like morphologies around the condensate periphery (Fig. 3 *A*–*C*); interestingly, we also observed significant aggregate accumulation around the nuclear periphery. This condensate surface accumulation of aggregates in the cytoplasm is seen in both fixed, light-activated samples (Fig. S5) and by live-cell imaging, where newly condensed p62 condensates accumulate small PolyQ aggregates on their interfaces on a timescale of minutes. The co-localization of these PolyQ aggregates to de novo condensates was quantified with a radial intensity plot, which exhibits a clear peak in the co-localized PolyQ aggregate signal close to the condensate periphery (Fig. 3 *B*).

To assess whether this rapid accumulation of PolyQ aggregates upon p62 condensation was due to the coarsening of existing aggregates, as opposed to the nucleation of new aggregates on the condensate surface, we measured the normalized bulk PolyQ aggregate intensity. The bulk intensity exhibited little to no change (Fig. S6 *A*), suggesting that PolyQ aggregates at the interface reflect the reorganization of small, existing aggregates that are already present before p62 condensation. In addition, this phenomenon was primarily observed in cells exhibiting a high number of small PolyQ aggregates (<5 *μ*m^2^) (Fig. S6 *B*). These findings suggest that the capacity for de novo p62 condensates to spatially reorganize cytoplasmic PolyQ aggregates depends on preferential attachment of small PolyQ aggregates to the condensate periphery.

To evaluate whether the aggregated material at the condensate interface would remain clustered, we performed repeated cycles of p62 condensate formation and dissolution. Aggregate clustering by p62 condensation followed by dissolution enhances coarsening of the interface-associated aggregates (Figs. 3 *C* and S7). This was supported by the appearance of persistent, coarsened aggregates after each condensation-dissolution cycle (Videos S1 and S2 in supporting material), shown as an increase in average aggregate size after successive cycles (Fig. 3 *D*). As additional cycles were performed, the rate of aggregate coarsening decreased, likely reflecting a limited capacity for continued interfacial recruitment of larger aggregates.

Video S1. Aggregate coarsening by condensation cycles

Video S2. Aggregate coarsening by condensation cycles near the nuclear boundary

### Large coarsened ubiquitinated aggregates inhibit further p62 condensation

**Figure 4:**
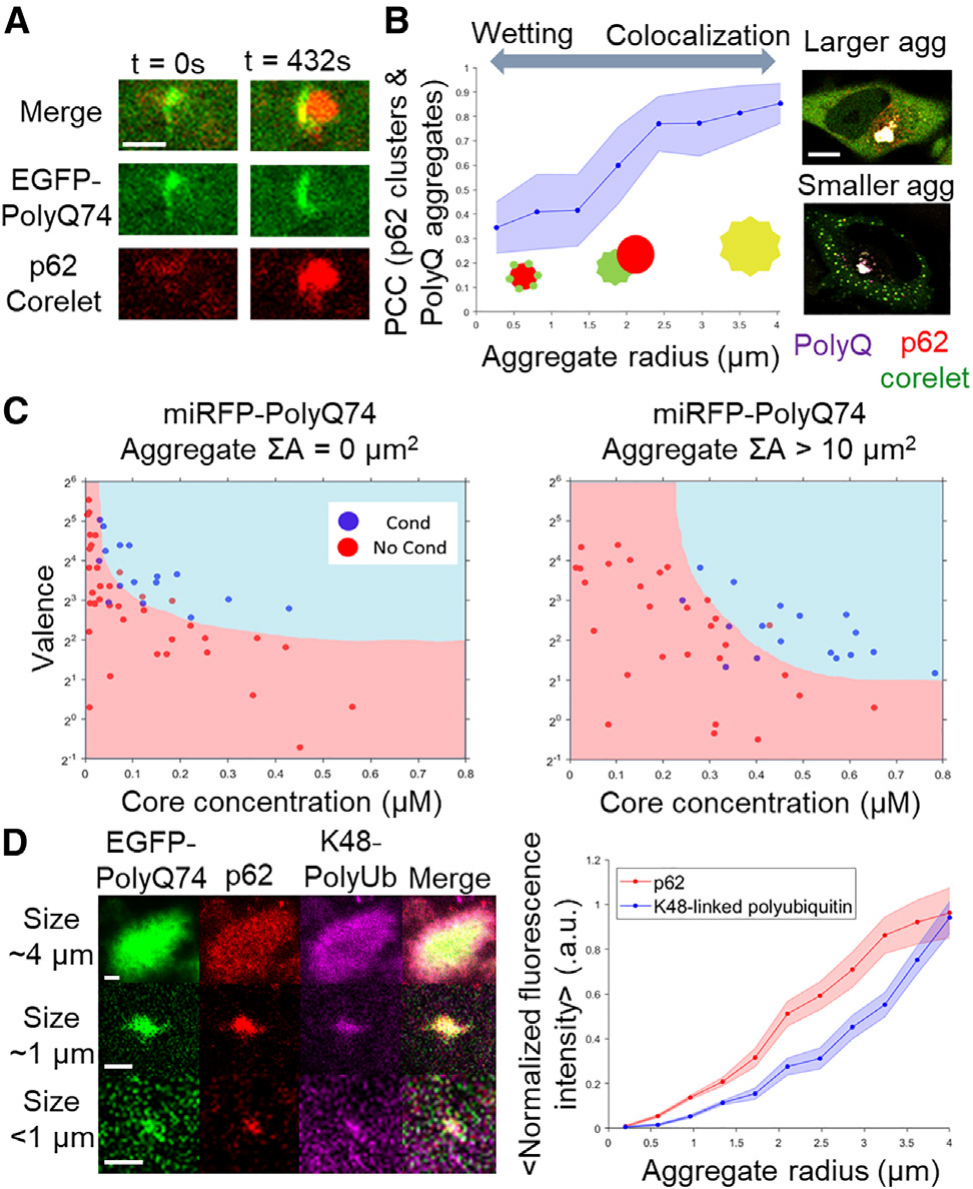
Large ubiquitinated aggregates recruit free p62 and suppress p62 condensation. (*A*) Light-inducible p62 corelet condensation on the surface of a PolyQ aggregate in a HeLa cell over the period of 7 min. Diagram of differential outcomes of p62 oligomerization in the presence of PolyQ aggregates. Scale bar, 2 *μ*m. (*B*) PCC between p62 clusters and PolyQ aggregates in physical contact for 47 pairs in 10 cells. Image of inducing p62 oligomerization in the presence of PolyQ aggregation of different sizes. Scale bar, 5 *μ*m. (*C*) Phase diagram of light-induced oligomerization of p62 in the presence of PolyQ expression, where the blue regime involves observation of multiple de novo spherical condensates and red involves an absence. Phase diagram of inducible p62 in the presence of PolyQ aggregate accumulation (>10 *μ*m^2^). (*D*) Representative images from immunofluorescent labeling of K48-linked polyubiquitin and endogenous p62 on PolyQ aggregates. Scale bar, 1 *μ*m. Integrated intensities of polyubiquitin and p62 signals normalized to respective maxima, for aggregates of various sizes in 11 cells. Error bars are based on mean ± SE.

To evaluate factors that might lead to reduced aggregate coarsening after repeated cycles, we specifically induced p62 condensation in the presence of larger cytoplasmic PolyQ aggregates. Interestingly, aggregates were capable of nucleating new p62 condensates directly on their surfaces (Fig. 4 *A*). To examine whether co-localization of oligomerized p62 and PolyQ aggregates is dependent on aggregate size, we measured the Pearson correlation coefficient (PCC) between p62 and PolyQ aggregate clusters of varying size (Fig. 4 *B*). Although p62 condensation of the smallest detectable aggregates resulted in PCC values of roughly 0.4, we unexpectedly found a trend of increasing PCC with aggregate radius. The largest aggregates exhibited near-perfect co-localization, strongly recruiting the oligomerized p62. We identified two distinct morphological outcomes for oligomerized p62 recruitment, with the small and medium-sized aggregates in contact with spherical condensates, whereas the largest aggregates exclusively favored p62 recruitment.

Given this PolyQ size-dependent co-localization behavior, we further examined the impact of large aggregates on p62 condensation. Cells with large aggregates had diminished de novo condensate formation (Fig. 4 *B*), even at total cellular concentrations expected to drive p62 phase separation. To quantify this change in phase behavior, we generated two distinct phase diagrams, one for cells that exhibit large pre-formed aggregates and one for cells that do not. A notable shift in the phase boundary is seen between the two phase diagrams (Fig. 4 *C*), reflecting an inhibition of condensate formation in the presence of large aggregates. Thus, extensive coarsening of PolyQ aggregates ultimately suppresses further p62 phase separation.

We hypothesized that the size-dependent changes in p62 co-localization might result from larger aggregates accumulating more ubiquitinated substrates, to which the UBA domain of p62 then binds, recruiting soluble p62. To test this, we labeled K48-linked polyubiquitin and endogenous p62 present in PolyQ aggregates by immunofluorescence and found an increase in integrated intensity with increasing aggregate size (Fig. 4 *D*). These observations suggested that, by accumulating more total ubiquitinated species, large cytoplasmic aggregates recruit more p62, decreasing the free pool of p62 available for new condensate formation. The interplay between p62 phase behavior and the extent of aggregate coarsening suggests a preference for enhanced coarsening in cells exhibiting predominantly small aggregates, whereas excessive aggregate coarsening and further ubiquitination suppresses additional p62 condensation.

## Discussion

The interplay between solid-like assemblies and liquid-like biomolecular condensates is thought to underlie a variety of dynamic regulatory processes in living cells (29,30,31,32,34,35,36,37,38). In the context of neurodegenerative diseases, the enrichment or absence of p62 in pathological aggregates has been suggested to strongly influence aggregation behavior (45,46). However, the role of p62 condensate formation in the dynamics of aggregation has remained elusive, due to the challenge of disentangling the biophysical determinants of condensation and aggregation, within the complex and time-varying (e.g., cell cycle) intracellular environment. In this study, we have addressed this challenge by adapting an optogenetic platform to rapidly and reversibly control p62 oligomerization and condensation, revealing an unexpected interface-driven coarsening of Huntingtin polyQ aggregates.

**Figure 5:**
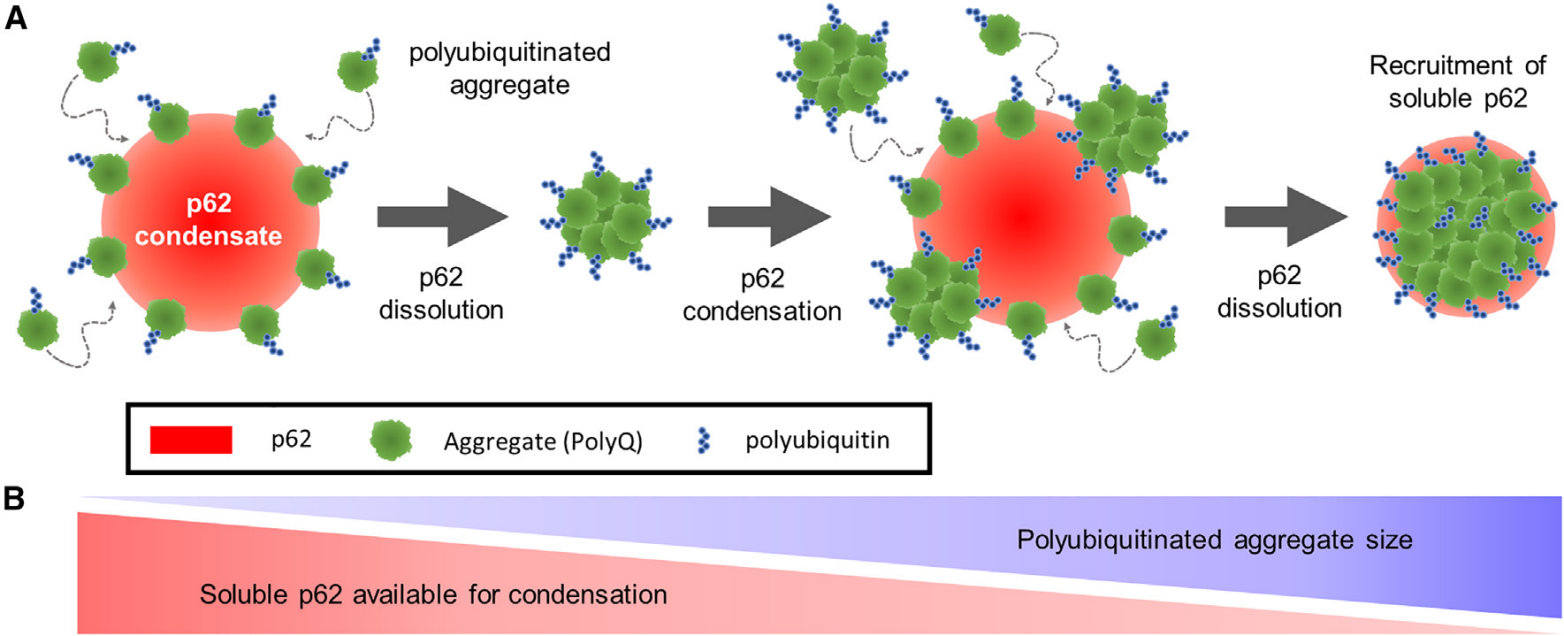
p62 oligomerization enhances PolyQ aggregate coarsening differentially based on aggregate size and resulting polyubiquitin accumulation. (*A*) Small polyubiquitinated PolyQ aggregates adhere to the interface of p62 condensates, where condensate dissolution compacts and coarsens attached aggregates. Large, coarsened aggregates recruit significant amounts of soluble p62. (*B*) Aggregate coarsening is enhanced by cycles of p62 condensation and dissolution but the recruitment of soluble p62 to large aggregates depletes the available pool of soluble p62 for condensation.

Our findings revealed that p62 condensation clusters cytoplasmic PolyQ aggregates onto the condensate interface. Moreover, upon condensate dissolution, the PolyQ aggregates are driven together, further increasing the average aggregate size (Fig. 5 *A*). Small biomolecular assemblies have previously been reported to preferentially assemble on the surface of germline P granules in developing *Caenorhabditis elegans* embryos (32). This process appears to stabilize P granules from further coarsening and is akin to Pickering emulsions described in nonliving systems (47). This effect is essentially the inverse of our findings: in contrast to surface-associated particles suppressing condensate coarsening, we find that condensates can enhance surface-associated aggregate coarsening. Our aggregate coarsening dynamics are similar to that seen upon the evaporation and dissolution of droplets containing colloidal particles, where large particle assemblies are driven together (48). The close resemblance between this behavior and our observations of condensate-mediated coarsening of PolyQ aggregates suggest that the interplay of aggregates and droplets in both living and nonliving systems may be governed by similar physical principles.

Protein aggregates, such as PolyQ aggregates, continually coarsen into larger sizes with the accumulation and transport of misfolded, aberrant proteins (49,50). The resulting broad distribution of PolyQ aggregate sizes (51) may play an important role in the clustering of aggregates to condensate interfaces. For example, we found that small aggregates are preferentially recruited to de novo p62 condensate interfaces, whereas the later presence of large aggregates ultimately inhibits condensation. The coarsening of small aggregates by recruitment onto p62 or other condensate surfaces could reflect protective benefits of forming larger aggregates over smaller oligomeric states (22,52,53); in the cases of α-synuclein in Parkinson’s, tau in Alzheimer’s, and Huntingtin polyQ in Huntington’s, small oligomeric assemblies have been suggested to be more toxic (54,55,56). The clustering of aggregates may also play a role in reducing the accessible surface area of aggregates to interfere with healthy, functional proteins. In contrast, the inhibition of further condensation by particularly large aggregates may reflect an upper limit of the cell’s ability to utilize condensates to modulate aggregate coarsening.

As larger aggregate sizes are generated in the cytoplasm, p62 concentration and ubiquitination also continue to play important roles in interface-mediated aggregate coarsening. We show that the intracellular concentration of multimerized p62 finely tunes the propensity for p62 to undergo condensation. Decreases in p62 concentration can result from autophagic or degradative processes that consume p62 (57), whereas increases in p62 concentration are common in cells with dysfunctional degradation or an increased cellular need for p62 (58). Gradual or rapid changes in p62 concentration due to cellular processes may influence aggregate coarsening rates through changes in p62 condensation propensity. Alongside p62 concentration, aggregate ubiquitination state determines its recognition by p62, which differentiates misfolded, aggregation-prone proteins by their accumulated ubiquitinated chain motifs (59). Our study showed that the larger the aggregate, the more K48-linked polyubiquitin. This supports our observation that the largest aggregates most strongly recruited soluble p62, which, in turn, inhibited p62 condensation (Fig. 5 *B*). These findings underscore how p62 condensation and pathological protein ubiquitination and aggregation reflect a subtle interplay wherein these processes can both promote and suppress one another, testing the limits of cellular mechanisms to minimize the pathological consequences of aggregation-prone proteins.

Our findings elucidate a new biophysical dimension of protein aggregation whose dynamics can be mediated by interfaces between liquid-like condensates and toxic solid-like aggregates. This concept is consistent with a broader emerging theme, with biomolecular condensate surfaces underlying diverse aspects of intracellular organization, from modulating condensate coarsening behavior (32), to driving co-localization of protein interaction networks (19,60,61), and the multi-step assembly of molecular complexes in the nucleolus (62). The physical mechanisms underlying the widespread interface-mediated coupling of different liquid and solid-like assemblies found throughout cells and tissues, and the consequences for physiology and disease, represent an exciting new frontier of cell biology.

## Author contributions

C.C., D.S.W.L., D.W.S., and C.P.B. designed research. D.W.S. provided additional reagents. C.C. performed research. C.C. and D.S.W.L. analyzed data. C.C. and C.P.B. wrote the paper.

## Supporting material

Document S1. Figures S1–S7

Document S2. Article plus supporting material
