# Supplementary material for: Condensate interfaces can accelerate protein aggregation
Source: Biophys J. 2023 Oct 13;123(11):1404–13. doi: 10.1016/j.bpj.2023.10.009 (PMC11163288; doi:10.1016/j.bpj.2023.10.009)
Supplement: Document S1. Figures S1–S7 [file mmc1.pdf]

Biophysical Journal, Volume *123*

Supplemental information

Condensate interfaces can accelerate protein aggregation

Chang-Hyun Choi, Daniel S.W. Lee, David W. Sanders, and Clifford P. Brangwynne

Supplemental Methods

Plasmids and Cloning

Genes of interest were amplified using CloneAmp HiFi PCR Premix (Takara Bio) with synthesized oligonucleotides (IDT), inserted into a lentiviral vector with the EF1α promoter, and transformed into Stellar cells (Clontech), from which single colonies were picked and grown in LB broth with ampicillin selection for miniprep (QIAGEN). polyQ aggregation construct EGFP-polyQ74 was an adaptation of pEGFP-Q74, a generous gift from David C. Rubinsztein (Addgene plasmid #40262) with re-insertion of complete N17 and C38 terminal residues to recover the Exon 1 sequence, reported in our previous study (41). miRFP-polyQ74 was generated by replacing the EGFP with miRFP670. EGFP-polyQ31 was generated because of variability in the repetitive polyQ lengths between single colonies, confirmed during sequencing for all plasmids (Genewiz). Corelet scaffold constructs iLID-EGFP-ft and iLID-ft were introduced in a previous study (32). The p62/SQSTM1 constructs, mCherry-p62, mCherry-p62ΔPB1, mCherry-p62ΔUBA were derived from HA-p62 a gift from Qing Zhong (Addgene plasmid # 28027). sspB-mCh-p62ΔPB1, sspB-mCh-p62ΔUBA were generated by amplifying the p62 gene with appropriate truncation from mCherry-p62ΔPB1 and mCherry-p62ΔUBA with ligation into an FM5 vector with sspB-mCh followed by a standard linker motif.

Lentiviral Transduction

All cells expressing constructs were stably transduced with lentivirus. The transfer plasmids, alongside VSVG and PSP helper plasmids were co-transfected using FuGENE HD Transfection Reagent (Promega) into the Lenti-X 293-T packaging line cultured in 6-well cell culture plates (Thermo Fisher) containing DMEM. After 48 hrs, the viral supernatant was collected using 0.45μm PES filters and stored in -80°C until use. Cells were reverse transduced by addition of viral media, followed by trypsinized cells quenched with DMEM, and replacement with fresh DMEM on the following day. polyQ aggregation experiments involved the addition of an appropriate volume of viral media to observe the onset of aggregation 3-days post-transduction. P62/Corelet experiments involved adding viral media at varying ratios simultaneously to individual wells, and then 1 day before experiments, pooling and replating for generating phase diagrams. P62 full-length and truncation experiments required adding a range of viral media between individual wells and pooling. Aggregate/p62/Corelet experiments involved transduction of polyQ for 2 days, then trypsinization and additional transduction with an appropriate ratio of p62/Corelet lentivirus to observe inducible p62 condensation. After 2 days post-transduction of p62/Corelet constructs into the aggregate-positive cells, cells were replated onto glass bottom plates for imaging.

Immunofluorescence protocol

Cells expressing constructs of interest were grown to 70% confluency in DMEM on 96-well glass bottom fibronectin-coated plates. To prepare for fixation, cells were washed with 4°C DPBS and fixed using a freshly-prepared 4% PFA solution in DPBS with a 15 minute incubation period. Afterwards, the PFA solution was removed and properly disposed of, followed by 3 washes in DPBS for 5 minutes each. Cells were then permeabilized using a 0.25% Triton-X PBS solution for 20 minutes, which after removal was followed by blocking for 1 hr at room temperature using a 0.1% Triton-X PBS solution with 10% normal goat serum (Vector Laboratories). Blocking solution was removed and followed by primary immunostaining overnight in 4℃ involved the following antibodies in blocking solution: anti-p62/SQSTM1 (1:400, MBL, PM045), anti-ubiquitin Lys48-specific (1:400, EMD Millipore, 05-1307). After washing 3 times for 10 minutes each with a 0.1% Triton-X DPBS solution, secondary immunostaining was performed for 90 minutes at room temperature using anti-rabbit Alexa Fluor 568 secondary antibody (Thermo Fisher, A-11036). After a final 3 washes of 10 minutes each, with 15 minute incubation to visualize the nucleus with 2mg/mL Hoechst dye in DPBS, the samples were immediately imaged in DBPS. Control samples lacking primary antibody were also labeled with secondary antibody to assess specificity.

Western blot protocol

HeLa cells stably transfected with p62 Corelet constructs, sspB-mCh-p62ΔPB and iLID-GFP-ft, were treated with 10 µm MG-132 (Sigma, 474787) for 6 hours to inhibit proteasomal degradation. In addition, a sample without MG-132 treatment was prepared as a control. Both samples were then trypsinized, quenched with DMEM, spun down, and then resuspended in DPBS for a second spin-down to remove residual trypsin/DMEM. Spun-down cells were then resuspended in DPBS with protease inhibitor and sonicated to generate cell lysates. The cell lysate protein concentrations were quantified by Bradford Assay (Millipore Sigma) and prepared in Novex NuPAGE LDS buffer (Invitrogen) with 75 mM DTT (Thermo Fisher). After denaturation at 100℃, 10 µg of protein from treated and untreated cells were loaded into a NuPAGE 4%–12% Bis-Tris protein gel, with Pageruler Prestained Protein Ladder (Invitrogen) in a separate well. The gel was then run in NuPAGE MOPS buffer (Invitrogen) at 125V for 90 min and transferred onto a PVDF membrane in NuPAGE transfer buffer (Invitrogen) at 25V for 1 hr. The membrane was then blocked in TBST with 5% non-fat dry milk (Nestle) for 3 hrs at 4℃. The membrane was then cut at 50 kDa to probe for both β-Actin and K48-linked ubiquitin. The bottom strip of the cut membrane was probed for β-Actin using rabbit anti-β-Actin antibody (1:10000 abcam, ab8227) at 1:10000 in blocking solution overnight at 4°C, while the remaining top strip was probed for K48-linked ubiquitin using rabbit K48-specific anti-ubiquitin antibody (1:5000, EMD Millipore, 05-1307) in blocking buffer overnight at 4°C. After washing in TBST, both membranes were probed with anti-rabbit HRP secondary antibody (1:10000, Jackson ImmunoResearch, 111-035-144) for 30 minutes at room temperature. Membranes were then treated with Clarity Western ECL Substrate (Bio-Rad) and the resulting chemiluminescent signal was imaged using a ChemiDoc MP Imaging System (BioRad).

Supplemental Figures

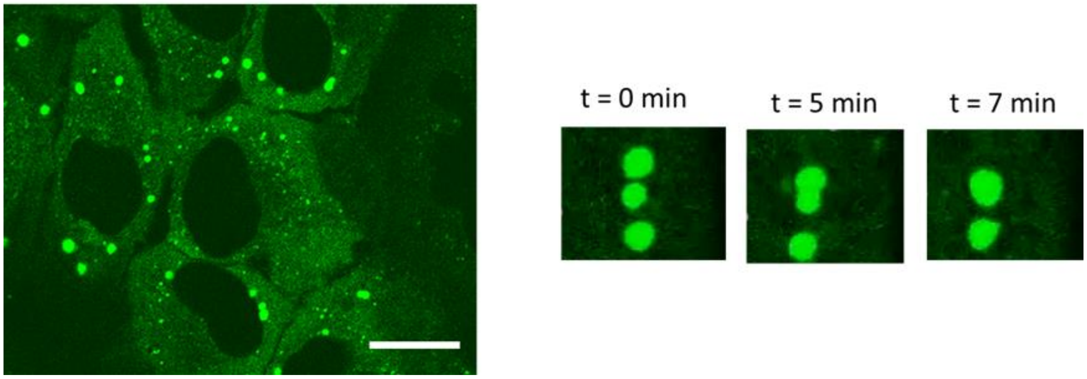

Figure S1 EGFP-p62 expressed in HeLa forming condensates. Zoomed-in fusion event of p62 condensates. Scale bar is 5 µm.

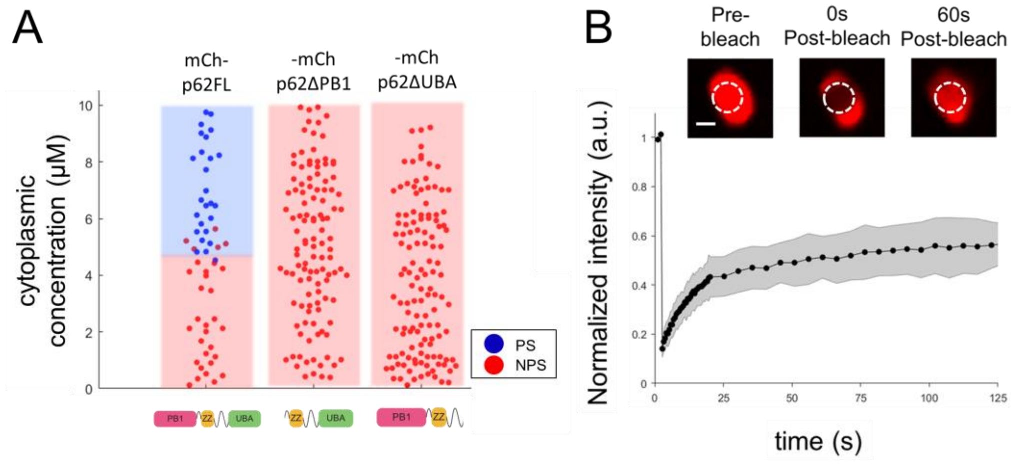

Figure S2 *(A)* Estimation of de novo p62 condensation for full-length mCherry-p62, and two mCherry-p62ΔPB1 and mCherry-p62ΔUBA constructs. Refer to Methods for threshold selection between PS “phase separated and NPS “non-phase separated” cells. *(B)* FRAP curve of a mCherry-p62 condensate measuring the recovery of fluorescence intensity in the ROI over 125 seconds. Scale bar is 1 µm.

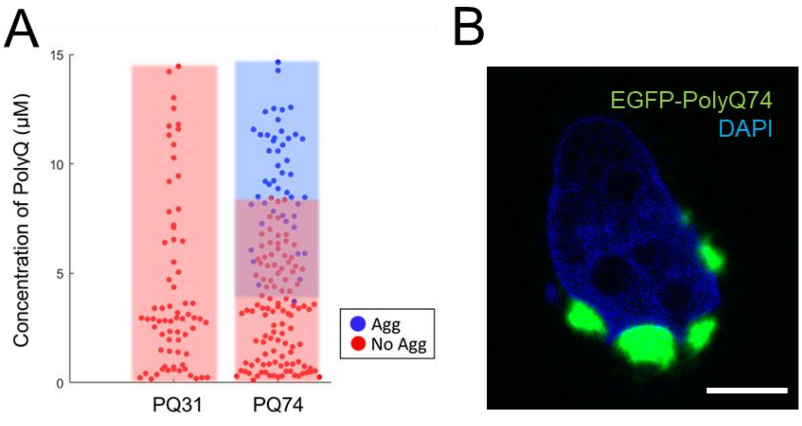

Figure S3 *(A)* Aggregation of individual cells with EGFP-PolyQ31 and EGFP-PolyQ74 expression. After 3 days of expression, the polyQ concentration in cells with and without aggregates were measured, where cells with aggregates were marked as a red point, and cells without aggregates as a blue point. The overlap of red and blue regimes reflects intermediate aggregate propensity over a range of concentration values in which we observed a mixed population of aggregated and non-aggregated cells. *(B)* Fixed HeLa cells expressing EGFP-PolyQ74 with nuclear DAPI staining, reflecting PolyQ aggregates as cytoplasmic assemblies. Scale bar is 5 µm.

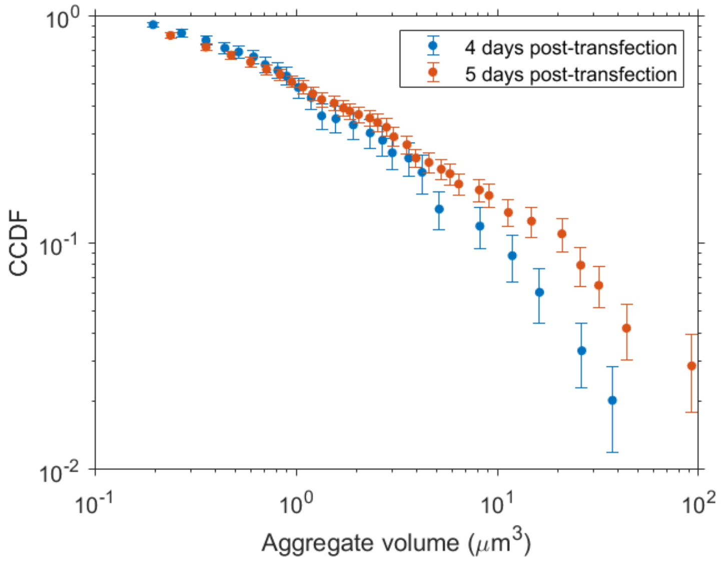

Figure S4 polyQ aggregate size distributions over time. Complementary Cumulative Distribution Function are averages of 34 cells day 4 post-transfection and 69 cells day 5 post-transfection.

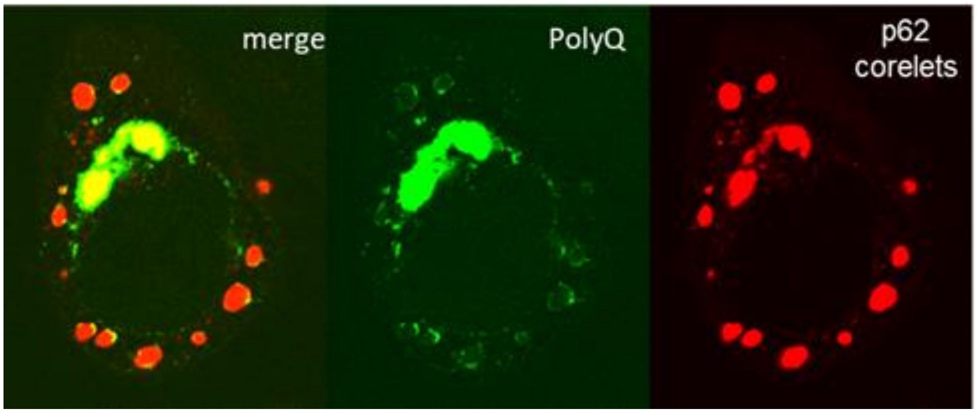

Figure S5 Ring-like accumulation of polyQ signal in fixed cells with induced p62 Corelet condensation. Blue light activation for 30 minutes using a LED plate, followed by fixation revealed a ring-like morphology of p62 droplet/aggregate interaction.

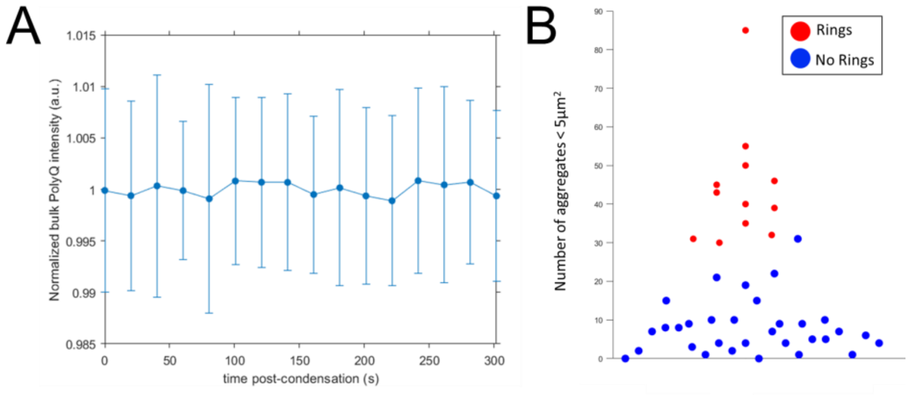

Figure S6 *(A)* polyQ aggregate fluorescence intensity normalized to the first frame over the immediate period post-condensation over 5 minutes for 10 cells. *(B)* Observation of ring-like accumulation of polyQ in cells exhibiting large numbers of small aggregates (< 5 µm^2) over those exhibiting fewer.

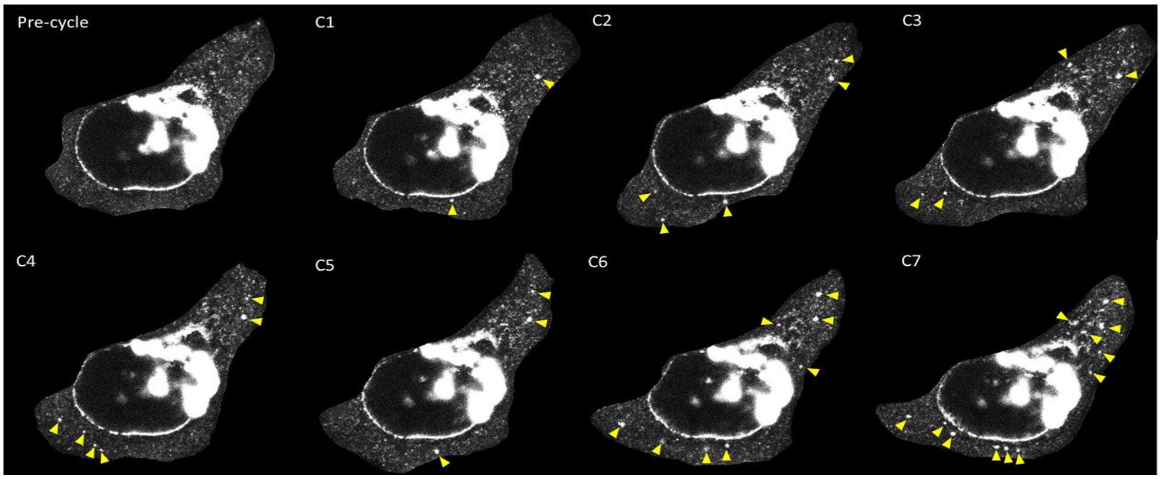

Figure S7 Example of polyQ aggregate coarsening in a cell following 7 cycles of p62 condensation and dissolution. Distinct, coarsened aggregates detectable by segmentation are labeled with yellow arrows.
